# Supplementary material for: Two Novel Yersinia pestis Bacteriophages with a Broad Host Range: Potential as Biocontrol Agents in Plague Natural Foci
Source: Viruses. 2022 Dec 8;14(12):2740. doi: 10.3390/v14122740 (PMC9785759; doi:10.3390/v14122740)
Supplement: Supplementary file 1 [file viruses-14-02740-s001.zip › Supplemental FigureS1.pdf]

(a)

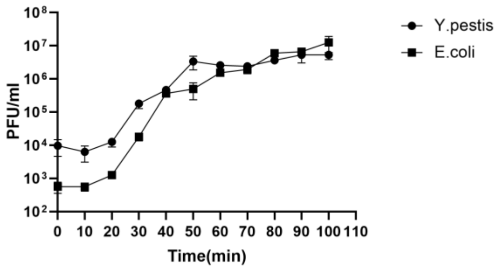

(b)

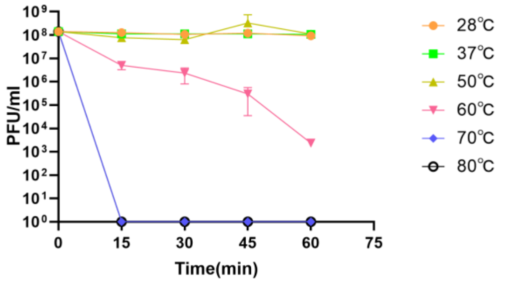

(c)

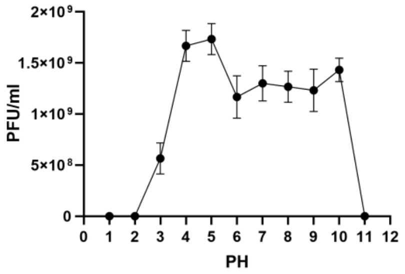

Supplemental Figure S1-A. Growth characteristics and stability test of MHS112 bacteriophage.
(a) Growth curve of MHS112 on *Y. pestis* (EV76) and *E.coli* (CMCC43201); Thermo Stability test (b) and pH stability test (c) of phage MHS112 on *Y. pestis* (EV76). The Y-axis shows the plaque-forming units per milliliter (PFU/mL). Each assay was performed as three repetitions, and the values represented are the means.

(a)

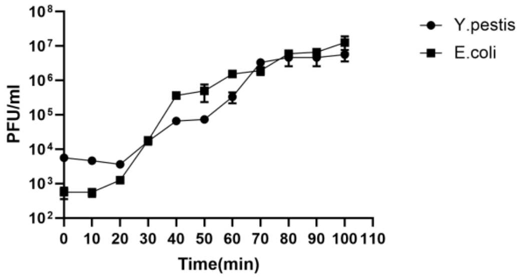

(b)

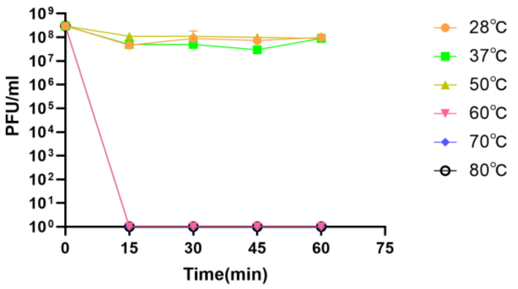

(c)

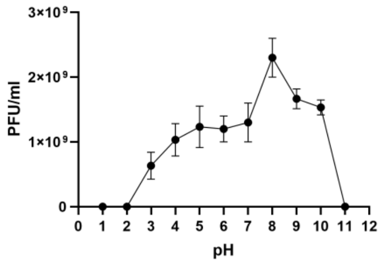

Supplemental Figure S1-B. Growth characteristics and stability test of GMS130 bacteriophage. (a) Growth curve of GMS130 on *Y. pestis* (EV76) and *E.coli* (CMCC43201); Thermo stability test (b) and pH stability test (c) of phage GMS130 on *Y. pestis* (EV76). The Y-axis shows the plaque-forming units per milliliter (PFU/mL). Each assay was performed as three repetitions, and the values represented are the means.
