# Supplementary material for: Two Novel Yersinia pestis Bacteriophages with a Broad Host Range: Potential as Biocontrol Agents in Plague Natural Foci
Source: Viruses. 2022 Dec 8;14(12):2740. doi: 10.3390/v14122740 (PMC9785759; doi:10.3390/v14122740)
Supplement: Supplementary file 1 [file viruses-14-02740-s001.zip › Supplemental Table S1.pdf]

Supplemental Table S1. Genomic Features of bacteriophages in this study

No.	phage	Isolation source of host bacteria	Genome size(kb)	Total CDSs	GC(%)	tRNA	Reference	GenBank Accession
1	*Yersinia* vB_YpeM_MHS112	*Yersinia pestis*	170.51	303	40.5	2	In this study	OP750247
2	*Yersinia* vB_YpeM_GMS130	*Yersinia pestis*	168.55	289	40.5	3	In this study	OP750248
3	*Aeromonas* phage 44RR2.8t	*Aeromonas salmonicida*	173.59	269	43.9	17	[1]	NC_005135
4	*Aeromonas* phage 65	*Aeromonas salmonicida*	235.23	454	37.2	16	[1]	NC_015251
5	*Aeromonas* phage Aeh1	*Aeromonas hydrophila*	233.23	375	42.8	23	[1]	NC_005260
6	*Enterobacteria* phage AR1	*Escherichia coli O157:H7*	167.44	291	35.3	10	[2]	NC_027983
7	*Enterobacteria* phage JS10	*Escherichia coli K12*	171.45	268	39.5	3	[3]	NC_012741
8	*Enterobacteria* phage JS98	*Escherichia coli*	170.52	269	39.5	3	[4]	NC_010105
9	*Enterobacteria* phage RB43	*Enterobacteriacea e*	180.500		43.2	1		NC_007023
10	*Enterobacteria* phage RB49	*Escherichia coli*	164.02	279	40.4	0	[1, 5]	NC_005066
11	*Enterobacteria* phage RB69	*Escherichia coli*	167.56	275	37.7	2	[1, 5]	NC_004928
12	*Enterobacteria* phage T4	*Escherichia coli*	168.9	288	35.3	8	[6]	NC_000866
13	*Vibrio* phage KVP40	*Vibrio parahaemolyticus*	244.83	415	42.6	29	[7]	NC_005083
14	*Vibrio* phage nt-1	*Vibrio natriegens HER1138*	247.51	405	41.3		[8, 9]	NC_021529
15	*Yersinia* phage JC221	*Yersinia pestis EV76-CN*	174.93	274	41.2		[10]	MN508817
16	*Yersinia* phage phiD1	*Yersinia pestis*	167.06	277	35.5		[11]	NC_027353
17	*Yersinia* phage PST		167.79	280	35.3	9	[12]	NC_027404
18	*Yersinia* phage PYPS2T	*Yersinia pseudotuberculosis*	169.604		35.4			NC_054944
19	*Yersinia* phage PYps5T	*Yersinia pseudotuberculosis*	169.38	267	35.4			MT828552
20	*Yersinia* phage PYps10T	*Yersinia pseudotuberculosis*	169.49	270	35.4			MT828553

21	*Yersinia* phage PYps11T	*Yersinia pseudotuberculosis*	169.49	268	35.4			MT515751
22	*Yersinia* phage PYps14T	*Yersinia pseudotuberculosis*	166.94	267	35.4			MT526905
23	*Yersinia* phage PYps15T	*Yersinia pseudotuberculosis*	166.93	267	35.4			MT515752
24	*Yersinia* phage PYps32T	*Yersinia pseudotuberculosis*	165.73	263	35.5			MT515753
25	*Yersinia* phage PYps35T	*Yersinia pseudotuberculosis*	166.97	264	35.4			MT515754
26	*Yersinia* phage PYps47T	*Yersinia pseudotuberculosis*	165.78	262	35.5			MT515755
27	*Yersinia* phage PYps55T	*Yersinia pseudotuberculosis*	167.71	267	35.4			MT515756
28	*Yersinia* phage v_YepM_ZN1*8*	*Yersinia pseudotuberculosis*	167.95	275	35.4	11		NC_054945
29	*Yersinia* phage fPS-2	*Yersinia pseudotuberculosis*	169.06		35.3		[13]	NC_054943
30	*Yersinia* phage fPS-65	*Yersinia pseudotuberculosis*	167.06		35.3		[13]	NC_055724
31	*Yersinia* phage fPS-90	*Yersinia pseudotuberculosis*	167.13		35.3		[13]	NC_055723
32	*Shigella* phage SP18	*Shigella sonnei*	170.61	289	40.4	3	[14]	NC_014595
33	*Enterobacteria* phage vB_EcoM_VR20	*Escherichia coli*	170.34	288	40.4	2	[15]	NC_028894
34	*Enterobacteria* phage vB_EcoM_VR25	*Escherichia coli*	170.82	295	40.4	2	[16]	NC_028925
35	*Enterobacteria* phage vB_EcoM_VR26	*Escherichia coli*	171.54	292	40.2	1	[16]	NC_028957
36	*Enterobacteria* phage vB_EcoM_VR7	*Escherichia coli*	169.29	294	40.3	1	[15]	NC_014792
37	*Escherichia* phage CJ20	*Escherichia coli*	169.88	307	40.4			MT533174
38	*Escherichia* phage DK-13	*Escherichia coli*	172.28	292	40.2			MT611523
39	*Escherichia* phage MN01	*enterotoxigenic Escherichia Coli*	172.58	290	40.4	2		MT129652
40	*Escherichia* phage P479	*Escherichia coli 187*	172.03	288	40.3		[17]	MW269952
41	*Escherichia* phage P896	*Escherichia coli*	170.66	293	40.4	2		OL451225
42	*Escherichia* phage PSD2001	*Escherichia coli*	172.18	285	40.3			OK254198
43	*Escherichia* phage UPEC01	*Escherichia coli*	169.80	307	40.4		[18]	MW233709

		S3Lug						
44	*Escherichia* phage UPEC07	*Escherichia coli*	170.00	308	40.3		[18]	MW250787
45	*Escherichia* phage vB_EcoM_RZ	*Escherichia coli str. K-12 substr. MG1655*	170.32	291	40.1			MW598459
46	*Escherichia virus* TH15	*Escherichia coli*	171.59		40.3			MT446392
47	*Escherichia virus* TH22	*Escherichia coli*	170.09		40.3			MT446396
48	*Escherichia virus* TH40	*Escherichia coli*	171.73		40.3			MT446411
49	*Escherichia virus* TH41	*Escherichia coli*	170.56		40.5			MT446412
50	*Escherichia virus* TH44	*Escherichia coli*	171.08		40.6			MT446415
51	*Escherichia virus* TH54	*Escherichia coli*	170.99		40.4			MT446420
52	*Escherichia virus* TH55	*Escherichia coli*	170.39		40.3			MT446421
53	*Escherichia virus* TH57	*Escherichia coli*	171.23		40.4			MT446422
54	*Vibrio* phage KVP20 major capsid protein	*Vibrio*					[19]	D83517
55	*Enterobacteria* phage TuIa major capsid protein (g23)	*Escherichia coli*					[1]	AF221994
56	*Enterobacteria* phage KC69 major capsid protein (g23)	*Escherichia coli*						AF221999

Reference:

1. Tetart F, Desplats C, Kutateladze M, Monod C, Ackermann HW, Krisch HM. Phylogeny of the major head and tail genes of the wide-ranging T4-type bacteriophages. J Bacteriol. 2001;183(1):358-66. Epub 2000/12/15. doi: 10.1128/JB.183.1.358-366.2001. PubMed PMID: 11114936; PubMed Central PMCID: PMCPMC94885.
2. Liao WC, Ng WV, Lin IH, Syu WJ, Liu TT, Chang CH. T4-Like genome organization of the Escherichia coli O157:H7 lytic phage AR1. J Virol. 2011;85(13):6567-78. Epub 2011/04/22. doi: 10.1128/JVI.02378-10. PubMed PMID: 21507986; PubMed Central PMCID: PMCPMC3126482.
3. Denou E, Bruttin A, Barretto C, Ngom-Bru C, Brussow H, Zuber S. T4 phages against Escherichia coli diarrhea: potential and problems. Virology. 2009;388(1):21-30. Epub 2009/04/03. doi: 10.1016/j.virol.2009.03.009. PubMed PMID: 19339031.
4. Chibani-Chennoufi S, Canchaya C, Bruttin A, Brussow H. Comparative genomics of the T4-Like Escherichia coli phage JS98: implications for the evolution of T4 phages. J Bacteriol. 2004;186(24):8276-86. Epub 2004/12/04. doi: 10.1128/JB.186.24.8276-8286.2004. PubMed PMID: 15576776; PubMed Central PMCID: PMCPMC532421.
5. Desplats C, Krisch HM. The diversity and evolution of the T4-type bacteriophages. Res Microbiol. 2003;154(4):259-67. Epub 2003/06/12. doi: 10.1016/S0923-2508(03)00069-X. PubMed PMID: 12798230.

6. Miller ES, Kutter E, Mosig G, Arisaka F, Kunisawa T, Ruger W. Bacteriophage T4 genome. Microbiol Mol Biol Rev. 2003;67(1):86-156, table of contents. Epub 2003/03/11. doi: 10.1128/MMBR.67.1.86-156.2003. PubMed PMID: 12626685; PubMed Central PMCID: PMCPMC150520.
7. Miller ES, Heidelberg JF, Eisen JA, Nelson WC, Durkin AS, Ciecko A, et al. Complete genome sequence of the broad-host-range vibriophage KVP40: comparative genomics of a T4-related bacteriophage. J Bacteriol. 2003;185(17):5220-33. Epub 2003/08/19. doi: 10.1128/JB.185.17.5220-5233.2003. PubMed PMID: 12923095; PubMed Central PMCID: PMCPMC180978.
8. Comeau AM, Bertrand C, Letarov A, Tetart F, Krisch HM. Modular architecture of the T4 phage superfamily: a conserved core genome and a plastic periphery. Virology. 2007;362(2):384-96. Epub 2007/02/10. doi: 10.1016/j.virol.2006.12.031. PubMed PMID: 17289101.
9. Comeau AM, Arbiol C, Krisch HM. Composite conserved promoter-terminator motifs (PeSLs) that mediate modular shuffling in the diverse T4-like myoviruses. Genome Biol Evol. 2014;6(7):1611-9. Epub 2014/06/22. doi: 10.1093/gbe/evu129. PubMed PMID: 24951563; PubMed Central PMCID: PMCPMC4122927.
10. Yuan Y, Xi H, Dai J, Zhong Y, Lu S, Wang T, et al. The characteristics and genome analysis of the novel Y. pestis phage JC221. Virus Res. 2020;283:197982. Epub 2020/04/22. doi: 10.1016/j.virusres.2020.197982. PubMed PMID: 32315702.
11. Skurnik M, Jaakkola S, Mattinen L, von Ossowski L, Nawaz A, Pajunen MI, et al. Bacteriophages fEV-1 and fD1 Infect Yersinia pestis. Viruses. 2021;13(7). Epub 2021/08/11. doi: 10.3390/v13071384. PubMed PMID: 34372590; PubMed Central PMCID: PMCPMC8309999.
12. Rashid MH, Revazishvili T, Dean T, Butani A, Verratti K, Bishop-Lilly KA, et al. A Yersinia pestis-specific, lytic phage preparation significantly reduces viable Y. pestis on various hard surfaces experimentally contaminated with the bacterium. Bacteriophage. 2012;2(3):168-77. Epub 2013/01/01. doi: 10.4161/bact.22240. PubMed PMID: 23275868; PubMed Central PMCID: PMCPMC3530526.
13. Salem M, Pajunen MI, Jun JW, Skurnik M. T4-like Bacteriophages Isolated from Pig Stools Infect Yersinia pseudotuberculosis and Yersinia pestis Using LPS and OmpF as Receptors. Viruses. 2021;13(2). Epub 2021/03/07. doi: 10.3390/v13020296. PubMed PMID: 33668618; PubMed Central PMCID: PMCPMC7917993.
14. Kim KH, Chang HW, Nam YD, Roh SW, Bae JW. Phenotypic characterization and genomic analysis of the Shigella sonnei bacteriophage SP18. J Microbiol. 2010;48(2):213-22. Epub 2010/05/04. doi: 10.1007/s12275-010-0055-4. PubMed PMID: 20437154.
15. Kaliniene L, Klausa V, Truncaite L. Low-temperature T4-like coliphages vB_EcoM-VR5, vB_EcoM-VR7 and vB_EcoM-VR20. Arch Virol. 2010;155(6):871-80. Epub 2010/04/03. doi: 10.1007/s00705-010-0656-6. PubMed PMID: 20361343.
16. Kaliniene L, Zajanckauskaite A, Simoliunas E, Truncaite L, Meskys R. Low-temperature bacterial viruses VR - a small but diverse group of E. coli phages. Arch Virol. 2015;160(5):1367-70. Epub 2015/03/11. doi: 10.1007/s00705-015-2388-0. PubMed PMID: 25753428.
17. Li P, Wang H, Li M, Qi W, Qi Z, Chen W, et al. Characterization and genome analysis of a broad lytic spectrum bacteriophage P479 against multidrug-resistant Escherichia coli. Virus Res. 2022;308:198628. Epub 2021/11/16. doi: 10.1016/j.virusres.2021.198628. PubMed PMID: 34780885.
18. Kazibwe G, Ndekezi C, Alinaitwe R, Alafi S, Nanteza A, Kimuda MP, et al. Genome Sequences of Bacteriophages UPEC01, UPEC03, UPEC06, and UPEC07 Infecting Avian Pathogenic Escherichia coli.

Microbiol Resour Announc. 2022;11(3):e0081121. Epub 2022/03/10. doi: 10.1128/mra.00811-21. PubMed PMID: 35262399; PubMed Central PMCID: PMCPMC8928767.
19. Matsuzaki S, Inoue T, Kuroda M, Kimura S, Tanaka S. Cloning and sequencing of major capsid protein (mcp) gene of a vibriophage, KVP20, possibly related to T-even coliphages. Gene. 1998;222(1):25-30. Epub 1998/11/14. doi: 10.1016/s0378-1119(98)00459-4. PubMed PMID: 9813227.
